# Pulmonary fibrosis model of mice induced by different administration methods of bleomycin

**DOI:** 10.1186/s12890-023-02349-z

**Published:** 2023-03-21

**Authors:** Aman Gul, Fangyong Yang, Cong Xie, Wenjing Du, Nabijan Mohammadtursun, Bin Wang, Jingjing Le, Jingcheng Dong

**Affiliations:** 1 Department of Integrative Medicine, Huashan Hospital, Fudan University, Shanghai, 200040 People’s Republic of China; 2 Institute of Integrated Traditional Chinese and Western Medicine, Fudan University, Shanghai, 200040 People’s Republic of China; 3 Central Laboratory, Xinjiang Medical University, Ürümqi, 830011 People’s Republic of China; 4 College of Xinjiang Uyghur Medicine, Hotan, 848000 People’s Republic of China; 5 Medical College of Hexi University, Zhangye, 734000 Gansu People’s Republic of China

**Keywords:** IPF mice model, Bleomycin, 18F-FDG, 3D ROI reconstruction

## Abstract

**Background:**

Idiopathic pulmonary fibrosis (IPF) is a chronic, progressive disease of the lung. How to build a typical human mimicking animal model has been a challenge. Thus, to reveal the mechanism and to make it useful for IPF clinical treatment, a different type of mice model and inspection methods are used to evaluate which one is applicable for the study of IPF.

**Method:**

69 Twelve-weeks-old C57BL/6 mice were divided into 3 type groups (n = 7 for each control group, n = 8 for each BLM-induced pulmonary fibrosis groups), as intraperitoneal injection, intratracheal administration, and intravenous administration of bleomycin (BLM) to initiate lung fibrosis. Changes of the lung function measured through mice Pulmonary function test (PFT). Morphological changes in mice were observed by PET/CT, Masson and Picro-Sirius staining, Transmission electron microscopy (TEM). Biochemical changes were tested by Enzyme-linked immunosorbent assay (Elisa).

**Results:**

PET/CT of BLM-receiving mice showed an increase in fibrotic consolidations and an increase in non-aerated lung area in BLM-treated mice compared with that in controls. TGF-b1, TNF-a, IL-6, GM-CSF in BALF and serum. PAI-1, HYP in the lung tissue of mice were significantly different in each BLM groups than those in the controls. The results of Masson staining in mice indicate that the lung tissues of all BLM received groups, the intratracheal groups, the intravenous groups, and the intraperitoneal groups have a higher degree of pulmonary septal thickening and collagen fiber consolidation compare to saline control. Picro-Sirius staining results are consistent with the results of Masson staining. Compared with the saline control group, the ratio of Col 1/Col 3 was significantly increased in each BLM group. TEM results found that in BLM group, type I alveolar epithelial cells were degenerated. Exfoliated endothelial cells were swelling, and type II alveolar epithelial cells were proliferated, the shape of the nucleus was irregular, and some tooth-like protrusions were seen.

**Conclusions:**

With three different methods of animal model construction, high dose of each show more compliable, and BLM can successfully induce animal models of pulmonary fibrosis, however, certain differences in the fibrosis formation sites of them three, and tail vein injection of BLM induced PF model is closer to the idiopathic pulmonary interstitial fibrosis.

**Supplementary Information:**

The online version contains supplementary material available at 10.1186/s12890-023-02349-z.

## Background

IPF is a chronic and progressive disease of the lung. It is characterized by the progressive scarring of lung tissues, the destruction of alveolar structures, the decreased lung compliance, and the impaired gas exchange, eventually leading to respiratory failure or even death [1]. High incidence is estimated in Europe and North America, with a rate of 2.8–18 new cases per 100,000 population per year, while in Asia and South America the incidence is lower, with a rate of 0.5–4.2 new cases per 100,000 population per year [2]. IPF is more common in males than females, with an age of onset after 50 years, a median diagnosis of 65 years, and a survival period of only 2–5 years after diagnosis [3]. IPF patients are not easy to be diagnosed in the early stage, and most patients are not diagnosed until the middle or late stage. Currently, there are almost no effective treatment methods. Therefore, the mortality rate is very high. The pathogenesis of IPF is still unclear [4]. At present, most researchers believe that it is related to the interaction of environmental exposure, infection, genetics, aging, and other factors [1, 5, 6]. Although no single animal model can summarize all the characteristics of human IPF, animal models still can partially reproduce the pathological manifestations of IPF, which is of great importance in the research of potentially effective treatment drugs [7]. The method of constructing an animal model is the groundwork for consecutive research. How to build a typical human mimicking animal model remains a challenge. Among various animal models of pulmonary fibrosis (BLM, FITC, silica, radiation, etc.), the BLM model is the most widely used mouse model with the best characteristics at present. BLM is a drug used to treat different types of neoplasm [8]. The most severe side effect of it is lung toxicity, which causes the remodeling of lung architecture and the loss of pulmonary function, rapidly leading to death. The experimental use of BLM is to induce IPF animal models. It leads to lung patchy parenchymal inflammation, epithelial cell injury with reactive hyperplasia, epithelial-mesenchymal transition, activation and differentiation of fibroblasts to myofibroblasts, and basement membrane and alveolar epithelium injures [9]. Positron emission tomography with 2-deoxy-2-[fluorine-18] fluoro-D glucose integrated with computed tomography 18F-FDG PET/CT, a tracer of tissue hypoxia, and useful for lung fibrosis monitoring is desperately missing [10, 11]. TGF-β1-dependent is upregulated [12, 13]. TNF-α-induced NF-κB activation promotes myofibroblast differentiation of LR-MSCs and exacerbates bleomycin-induced pulmonary fibrosis [14]. Suppression of plasminogen activator inhibitor-1 (PAI-1) by RNA interference attenuates pulmonary fibrosis [15]. Overexpression of collagen is a hallmark of organ fibrosis. The overexpression of collagen in fibrotic lungs is a key factor in tissue dysfunction. In patients with normal lungs and those with other pulmonary diseases, collagen type ratios were normal. In the fibrotic lungs from patients with adult respiratory distress syndrome, a shift was found in the ratio of Col I–Col 3 from the normal value of 2:1 to a mean value of 3.4:1[16]. The gene expression of both Col I and Col 3 after treatment with TGF − β was abolished by pirfenidone or rapamycin, while the most definitive response to the combined treatment of pirfenidone and rapamycin after TGF − β was found in Col3 [17].

Thus, to reveal the mechanism and to make it useful for clinical treatment for IPF, this study aimed to use a different type of IPF animal model and use many inspection methods to evaluate which one is more applicable for the study of IPF. Direct aspiration into the lungs and systemic delivery of BLM in mice was induced in pulmonary fibrosis. In the past, the rather commonly used intratracheal injection model fibrosis lesions were mainly distributed around the bronchi and bronchioles, which was inconsistent with the IPF lesions mainly distributed under the pleura [18]. And the incidence of complications such as intratracheal asphyxia and pulmonary infection was high. In recent years, the mouse model of low-dose multiple tail vein injections of BLM has been used to observe the efficacy of anti-pulmonary fibrosis drugs. This study compared tail vein injections of BLM with the intraperitoneal injection model and the intratracheal injection model to explore the lungs differences of each model and the characteristics of fibrosis.

## Methods

### Reagents

The enzyme-linked immunosorbent assay (ELISA) kits in this study were TNF-α (cat #: MEC1003), IL-6(cat #: MEC1008), GM-CSF (cat #: MEC1004), and TGF-β (cat #: MEC1012) were purchased from ANOGEN (Mississauga, Ontario, CANADA); The PAI-1 ELISA kit was purchased from Molecular Innovations (Novi, MI). HYP-detection kit (cat #: A030-1-1) was purchased from Njjcbio, China, Sirius red stain (G1018; Service bio).

### Animals

69 C57BL/6 mice (12 weeks old) were purchased from Shanghai SLAC Laboratory Animal Co., Ltd (SCXK2018-0003) and raised in a pathogen-free rodent facility according to the procedures approved (2021-01-SYXK-DJC-002; Additional file 1) by the Committee on the Ethics of Animal Experiments of Fudan University. The mice were housed in separate stainless-steel cages (six mice per cage) in a temperature-controlled environment (20–24 °C) on 12 h light–dark cycles with unrestricted access to food and water. All procedures of this study were followed by the “Experimental Animal Ordinance” of Fudan University. After adaptive feeding for 1 week, during the construction of the animal model with BLM (Hanhui Pharmaceutical Co., Ltd. Shanghai, China), we used 1% pentobarbital sodium, to anesthetize the mice via intraperitoneal injection 0.15 ml/20 g, while PET/CT process, after one hour of injection of 5 MBq [^18^F]-FDG mice were anesthetized with isoflurane, in an induction chamber, and the anesthesia machine was set to 1.5% isoflurane with a flow rate of 4.5 L/min oxygen. Observe the reduction of animals’ breathing (the percentage of isoflurane can be adjusted ± 0.5% depending on the susceptibility of the animals to isoflurane anesthesia). For lung function testing we used 1% pentobarbital sodium via intraperitoneal injection of 0.15 ml/20 g to the mice and we used a surgery board during tracheotomy (at approximately 70° from horizontal) [19]. After 1 week of adaptive feeding, the mice were randomly divided into nine groups, saline intraperitoneal (100 μl) control group, IPC, intraperitoneal injection of BLM low dose (20 mg/kg) group, IPL, high dose(50 mg/kg) group, IPH, saline intratracheal administration (50 μl) control group, ITC, intratracheal administration of BLM low dose (3 mg/kg) group, ITL, high dose (5 mg/kg) group, ITH, saline tail vein injection(100 μl) control group, IVC, intravenous administration (tail vein) of BLM low dose(10 mg/kg) group, IVL, high dose (20 mg/kg) group, IVH. For intraperitoneal BLM group, after sanitized with 75% of alcohol, we injected 20 mg/kg and 50 mg/kg for low dose (IPL) and high dose group (IPH), respectively, with constant volume of 100 μl, 100 μl saline for control repeated 7 days. For intratracheal administration of BLM, nonsurgical transoral instillation of BLM into mice lung method was used [19]. 2% sodium pentobarbital was prepared for anesthesia and injected into the abdominal cavity according to the corresponding dose of the mice's body weight (50 mg/kg). After anesthesia, mice lied supine on a fixed table with the limbs fixed, and the neck is shaved. After disinfection, laryngoscopes and LED light was used to make sure the BLM could accurately be perfused into trachea. In the low (ITL) and high dose (ITH) group, mice were injected with 3 mg/kg, 5 mg/kg BLM dissolved in 50 μl of saline, respectively, while in the control group mice were injected with an equal volume of saline. Tail vein injection BLM mouse model produced as followed: Put the mouse into a mouse fixer [20] (mouse injection cone with restrainer and LED GLOBALEBIO GEGD-Q9G), expose the tail, disinfect with 75% alcohol, and expose the tail vein. In the low dose (IVL) and high dose (IVH) group, mice were given BLM injection at 10 mg/kg and 20 mg/kg, respectively, with a 1 ml syringe (BLM was dissolved with normal saline at a concentration of 2 mg/ml) for 7 days; In the saline control group, mice were injected with 5 ml/kg normal saline through the tail vein for 7 days.

### Survival status and body quality testing

During the experiment, the activity, changes in the body weight, eating, drinking, fur, and survival status of the mice in each group were observed.

### PET/CT imaging and analysis

At day 25–26 animals were received successive PET/CT labelled with [18^F^]-FDG [10] after anesthetized through isoflurane (1.5%) inhalation for intraperitoneal injection of 5 MBq of 18^F^-FDG PET/CT 60 min before imaging. Mice were then maintained under anesthesia (1.5%) and placed on an imaging heated bed inside an Inveon MM PET (SIEMENS, JAPAN).

### Lung function test in mice

All the mice were anesthetized with an intraperitoneal injection of 1% pentobarbital (0.15 ml/20 g), and tracheostomy was conducted, as previously described [21], with a standard catheter. All the mice were tracheostomized and placed in a forced pulmonary function testing (PFT) system (Buxco, NY, USA) with FinePointe and FinePoint Control Panel software in the supine position on the bed within the plethysmograph, which simulated clinical pulmonary testing routinely performed on humans.

### Leukocyte classification and counts of bronchoalveolar lavage fluid (BALF)

BALF was collected by endotracheal intubation with 300 μl ice-cold PBS twice and then centrifuged at 500 g for 10 min at 4 °C. The supernatants were used for further ELISA. The total cells were resuspended with 50 μl PBS and counted via the Mindray BC-5000Vet automated hematology analyzer (Mindray, Shenzhen, CHN). (See Fig. 6).

### The indexes related to inflammation, fibrosis in BALF, serum, and lung tissue were determined by ELISA

The enzyme-linked immunosorbent assay (ELISA) kits in this study determined TGF-β1, TNF-α, IL-6 and GM-CSF antigen in BALF and Serum. The PAI-1 and HYP antigen in lung tissue were determined by using their ELISA kit.

### Histopathological tests

Histopathological Examination For histological analysis, pulmonary tissues were fixed with 4% paraformaldehyde and embedded in paraffin. The paraffin blocks were cut at 5 μm using a microtome. Sections stained against Masson Trichrome [14]. Collagen deposition of the sections were observed and assessed with an optical microscope and quantified with Image-Pro Plus6.0 software. PSR staining and quantification [22–24] were used in this test. The tissue sections were deparaffinized and rehydrated in a graded ethanol series and then incubated for 1 h in PSR staining. The stained sections were analyzed by using an ECLIPSE Ci-L/Ci-E Clinical Upright Microscope (Nikon; magnification, ×200) with a linear polarizer. To avoid missing any details, the filter was tilted to an angle between 0 and 90 until the birefringence became evident and the background became completely black. The focus was then corrected once more [23]. The halogen lamp intensity and exposure time were constant within each image. Under polarized light, Collagen type 1(also known as collagen alpha, Col1A1, and alpha-1 type 1 collagen, Col 1) appeared to red or yellow with strong birefringence, while Collagen type 3 (Col 3) was green with weak birefringence. The areas of Col 1 and 3 staining were analyzed by using Image Pro Plus 6.0 (Media Cybernetics, Inc.).

### Transmission electron microscopy images

Blocks were rinsed overnight in 0.1 M phosphate buffer (350 mOsm, pH 7.4) and fixed for two hours in osmium tetroxide (1% osmium tetroxide in 0.125 sodium cacodylate buffer, 400 mOsm, pH 7.4). Then the samples were passed through stepwise dehydration in increasing concentrations of ethanol (50–100%), rinsed with propylene oxide, and embedded in Araldite. Blocks were then cut into ultrathin Sections (50–70 nm) and contrast stained with saturated uranyl acetate and bismuth subnitrate. Sections were examined at an accelerating voltage of 60 kV 80 by using a Zeiss EM 10 C transformation electron microscope (HITACHI H-7500). Micrographs of a carbon grating replica were taken for calibration [25, 26].

### Statistical analysis

GraphPad Prism 8.0 statistical software was used for statistical processing of the related experimental data, all results flowed x ± SD. Normality tests and homogeneity of variance tests were conducted for the measurement data of each group. One-way ANOVA was used for the data in line with normal distribution and homogeneity of variance, and the Tukey test was used for pairwise comparison between multiple groups. *p* < 0.05 was considered statistically significant (*p < 0.05, **p < 0.01, ***p < 0.001, ****p < 0.0001).

## Results

**Fig. 1:**
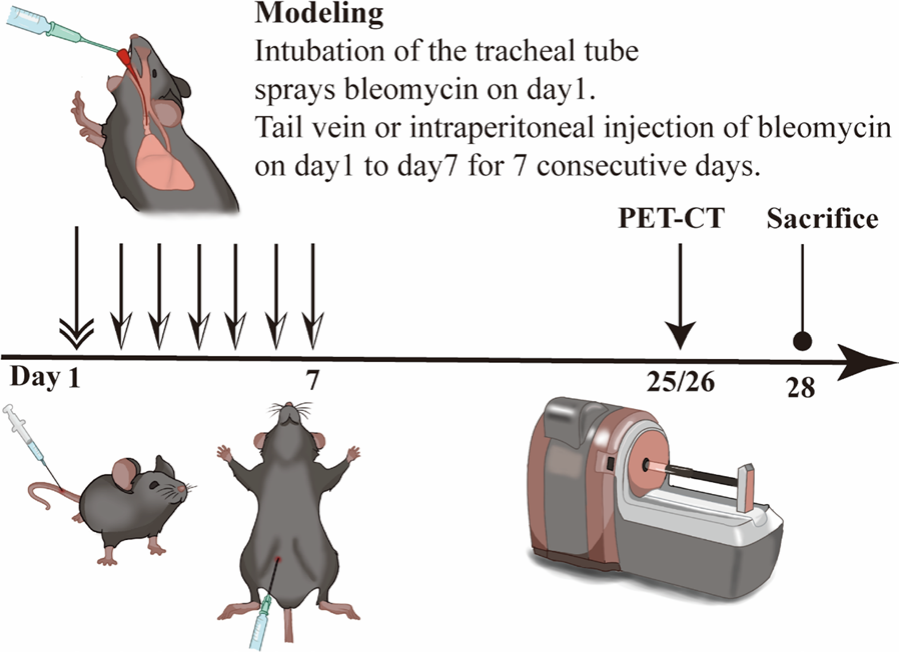
The timeline of the BLM-induced PF model construction

The timeline of the IPF model construction is shown in Fig. 1.

### Survival status and body quality testing

**Fig. 2:**
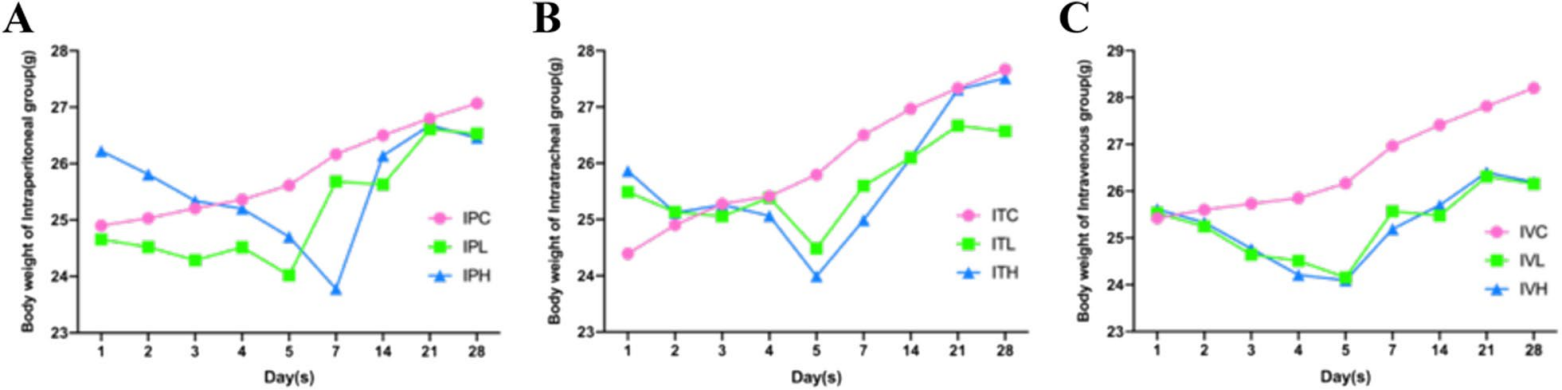
Body weight changes in each type of BLM administration group

Observation of the living conditions of the experimental mice in the three saline group had brighter fur, agile, lively, stable breathing, a normal diet, and gradually increased body weight without death (Fig. 2). The fur of the mice in the BLM group is dull. In the first 5 days of the intratracheal administration of the BLM group, there was less activity, slow response, shortness of breath, and often accompanied by coughing. In intraperitoneal injection of BLM group and tail vein injection group, the above symptoms were mild without coughing. After 5 days, the mice gradually recovered and their body mass increased, but their growth was slower than that of the saline control group. Analysis of mortality and cause of death of mice in each group in the normal group and three control groups, the mice had a good appetite for activity, and their fur was smooth and shiny, and their body mass continued to rise. There was no death, with zero fatality rate. The appetite activity of the mice in BLM groups was significantly reduced, with the fur less shiny, the spirits being lethargic, and the bodyweight decreasing significantly within 1–7 days of the experiment. After that, the body mass remained or slightly rebounded. The IVC, IVL, IVH mice of the tail vein group injected local skin with different degrees of swelling and necrosis, and different degrees of abdominal distension and slowness of movement can be observed in intraperitoneal group. ITL, ITH mice of the BLM group showed shortness of breath, coughing, and noisy (Table 1).

**Table 1:** Evaluation of IPF degree by 3D reconstruction image of 3D ROI segmentation of lung PET CT images

Group	IPC	IPL	IPH	ITC	ITL	ITH	IVC	IVL	IVH
No IPF	7	5	4	7	2	0	7	4	3
Mild IPF	0	2	2	0	1	0	0	1	1
Moderate IPF	0	1	2	0	4	2	0	2	2
Severe IPF	0	0	0	0	1	6	0	1	2

### PET/CT imaging and analysis

**Fig. 3:**
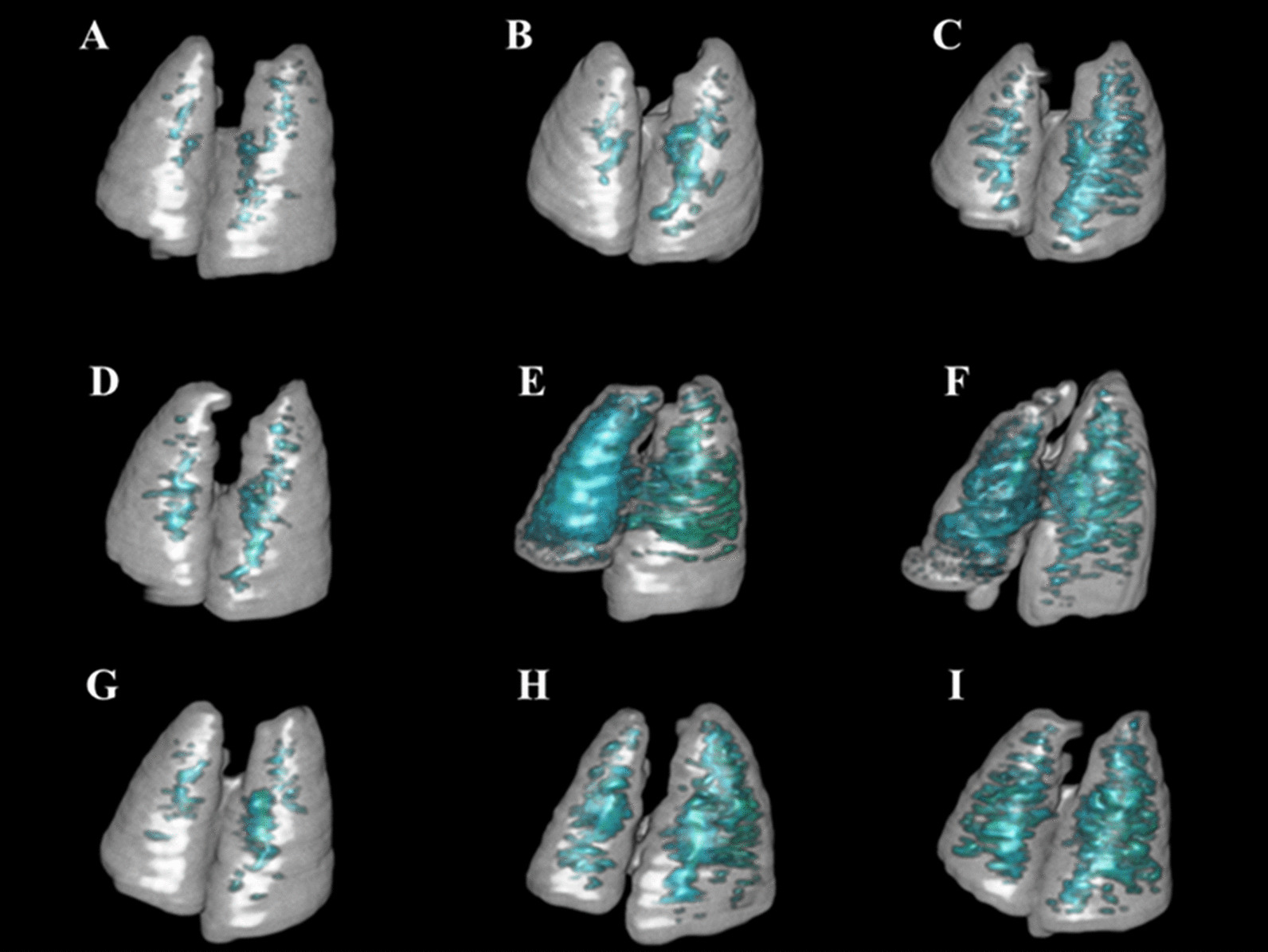
3D reconstruction image of 3D ROI segmentation of lung CT images. Representative 3D reconstruction of lung 3D ROI segmentation of lung CT images of Saline control and BLM-receiving mice at D26. Blue represented high-density lung areas (− 100 to 300 HU) which were the representative of non-aerated lungs. Grey represented normal density lung areas (− 800 to − 100 HU) which were the representative of aerated lungs. **A** IPC, **D** ITC, **G** IVC are those in saline administration controls, BLM lower dose groups **B** IPL, **E** ITL, **H** IVL and high dose groups **C** IPH, **F** ITH, **I** IVH, showed an increase in non-aerated lung area

**Fig. 4:**
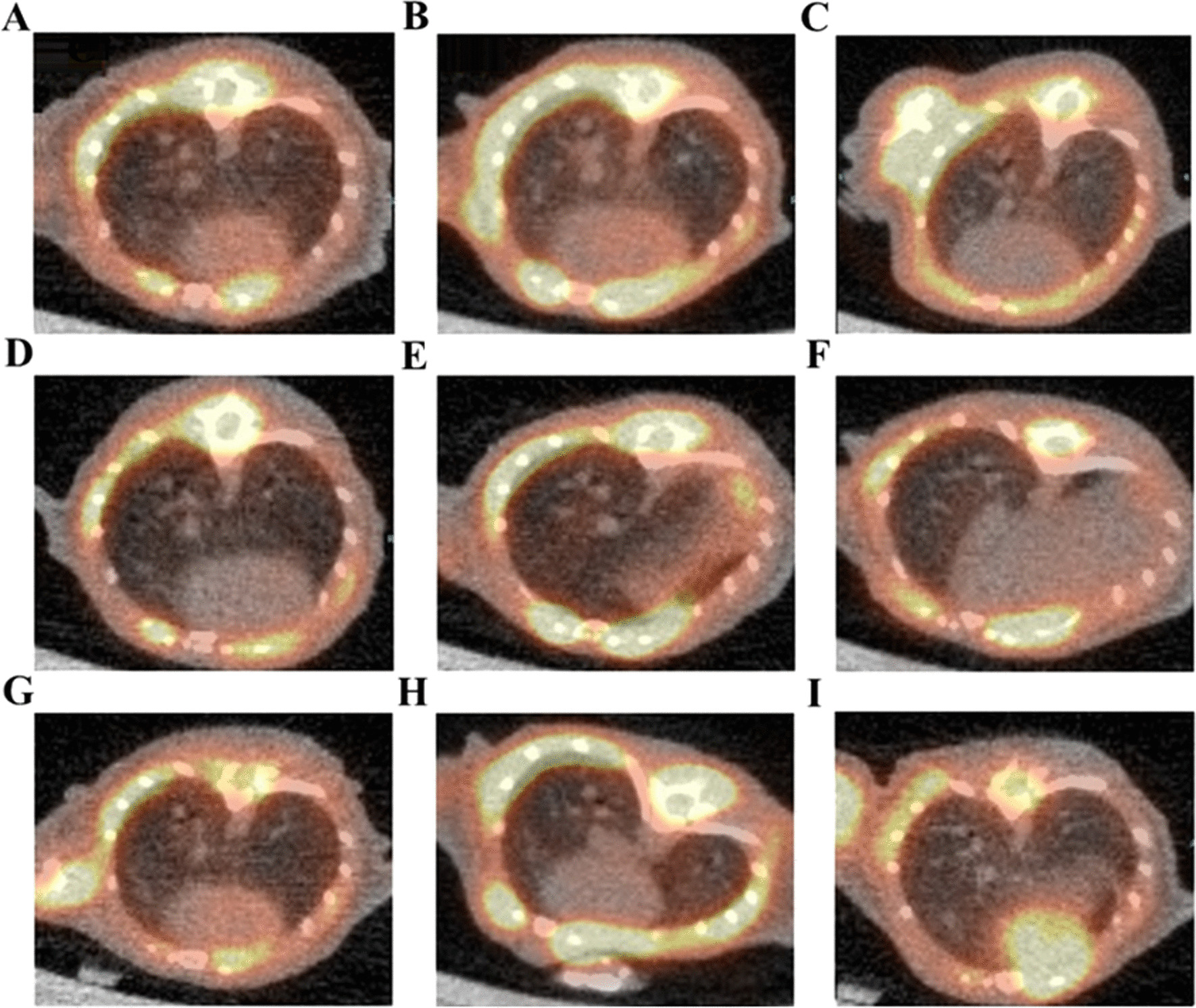
PET/CT images of each group BLM induced PF lung. Represented in three types BLM-receiving mice at D25-26. 18F-FDG uptake increased in BLM-induced PF groups. **A** IPC, **D** ITC, **G** IVC are those saline administration controls, BLM low dose groups **B** IPL, **E** ITL, **H** IVL and high dose groups **C** IPH, **F** ITH, **I** IVH

At day 25–26 animals were received successive PET/CT with [10]18^F^-FDG. After anesthetized through isoflurane (1.5%) inhalation for intraperitoneal injection of 5 MBq of 18^F^-FDG PET/CT 60 min before imaging. Mice were then maintained under anesthesia (1.5%) and placed on an imaging heated bed inside an Inveon MM PET (SIEMENS, JAPAN), shown in Figs. 3 and 4. Lung CT of BLM-receiving mice at D26 showed an increase in fibrotic consolidations compared with those in saline control mice (Fig. 3 left panel A-IPC, D-ITC, G-IVC), CT showed an increase in non-aerated lung area (Fig. 3) in BLM-treated mice compared with that in controls (Fig. 3 middle panel B-IPL, E-ITL, H-IVL, and right panel C-IPH, F-ITH, I-IVH). IPF degree Evaluated by 3D reconstruction image of 3D ROI segmentation of lung CT images, compared with that in saline control group mice, the high-density lung area of receiving BLM increased significantly, and the high-density lung area also increased with the dose of BLM, obviously increased (Figs. 3 and 4).

### Lung function

**Fig. 5:**
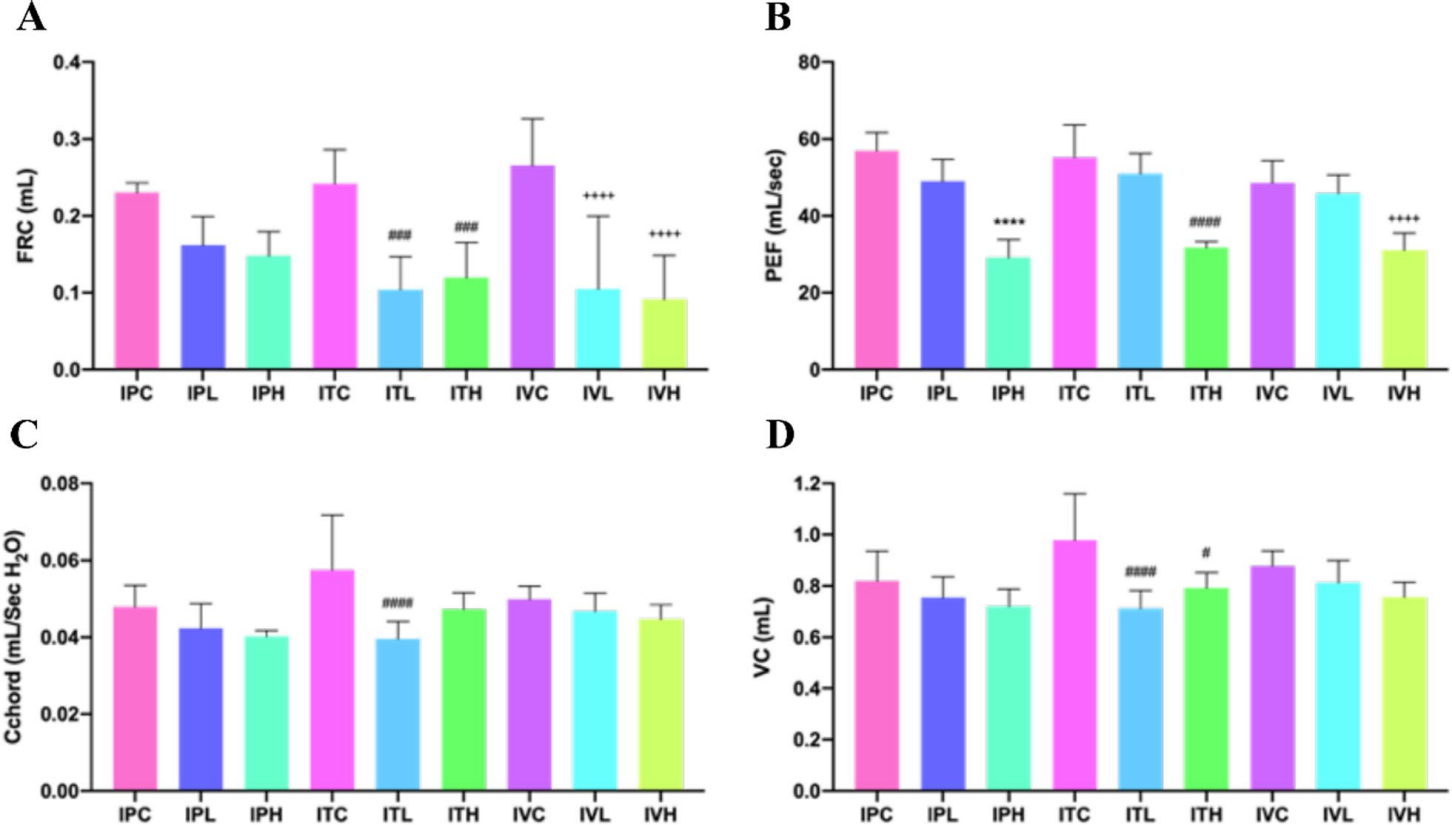
Pulmonary function changes and its analysis in each group. *p < 0.05, **p < 0.01, ***p < 0.001, ****p < 0.0001, compared to the IPC; ^#^*p* < 0.05, ^##^*p* < 0.01, ^###^*p* < 0.001, ^####^*p* < 0.0001, compared to the ITC; ^+^*p* < 0.05, ^++^*p* < 0.01, ^+++^*p* < 0.001, ^++++^*p* < 0.0001, compared to the IVC. The parameters included in pulmonary function test analysis are presented in Figure. **A** The FRC (functional residual capacity) showed a down-regulated trend in all groups, compared to that in the Saline group, while the FRC value significantly decreased in ITL, ITH, ^###^*p* < *0.001* compared with ITC and IVL, IVH, ^++++^*p* < *0.0001*compared with IVC; **B** PEF(Peak Expiratory Flow)showed a down-regulated trend in all groups, compared to that in the Saline group, while the PEF value significantly decreased in IPH, ^****^p < *0.0001*,ITH,^####^*p* < *0.0001*, IVH, ^++++^*p* < *0.0001*compared with IPC,ITC and IVC, respectively; **C** Cchord (Chord Compliance, between 0 and 10 cm H_2_O) showed a down-regulated trend in all groups, compared to that in the Saline group, the Cchord value significantly decreased in ITL,^####^*p* < *0.0001*, compared with ITC; **D** VC (Vital capacity) were significantly decreased in ITL, ^###^*p* < *0.0001*, and ITH, ^#^*p* < 0.05, compare to ITC

Pulmonary function changed in each group. The parameters included in pulmonary function test analysis are presented in Fig. 5. The FRC (functional residual capacity), PEF (Peak Expiratory Flow), Cchord (Chord compliance), and VC (Vital capacity) showed a down-regulated trend in all BLM groups, compared to those in the saline control. The pulmonary function was decreased in all model groups. Pulmonary function of intratracheal administration of BLM decreased seriously among these three groups.

### The indexes related to inflammation, fibrosis in BALF, serum, and lung tissue were determined by ELISA results

**Fig. 6:**
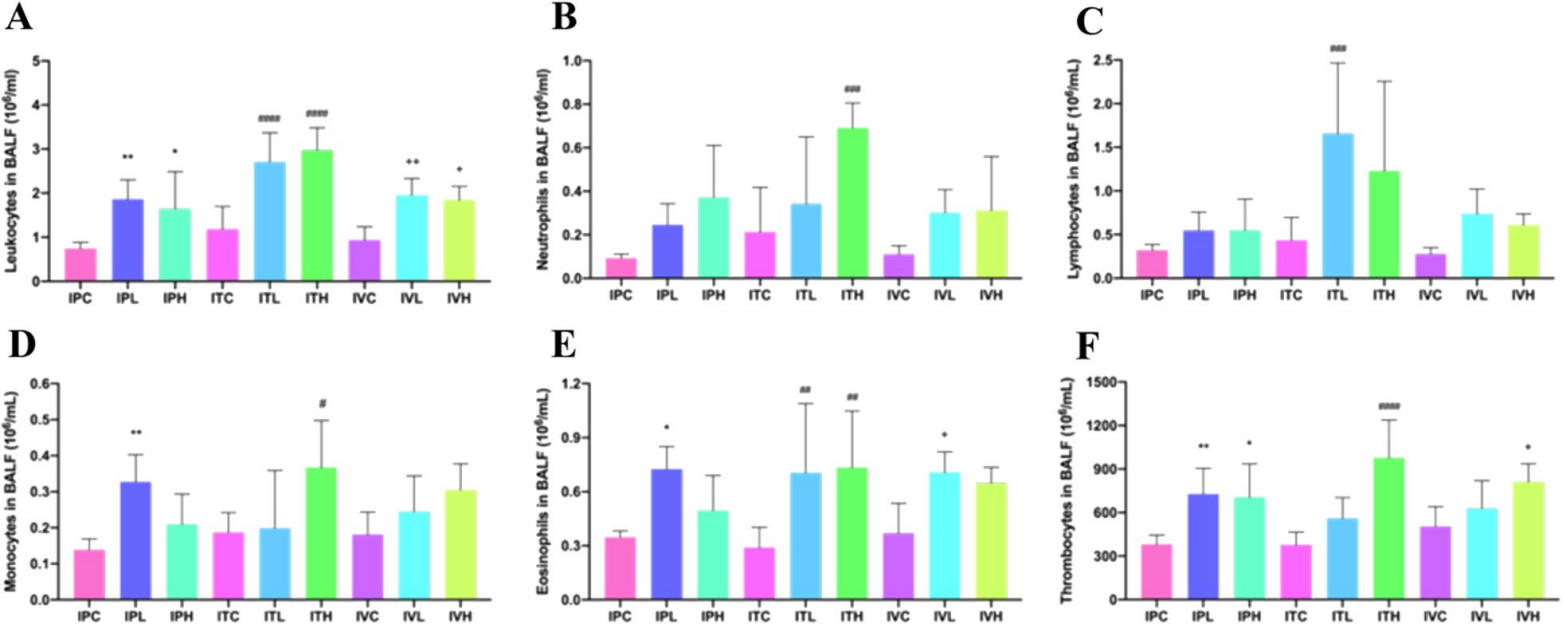
Cell counts in BALF. *p < 0.05, **p < 0.01, ***p < 0.001, ****p < 0.0001, compared to the IPC; ^#^*p* < 0.05, ^##^*p* < 0.01, ^###^*p* < 0.001, ^####^*p* < 0.0001, compared to the ITC; ^+^*p* < 0.05, ^++^*p* < 0.01, ^+++^*p* < 0.001,.^++++^*p* < 0.0001, compared to the IVC. The counts of inflammatory cells in BALF of each group of mice. Data were presented in Figure. The counts of inflammatory cells as Leukocytes, Lymphocytes, Monocytes, Neutrophils, Eosinophils, and Thrombocytes showed an up-regulated trend in all groups, compared to those in the saline administration control group. **A** The Leukocytes showed an up-regulated trend in all groups, compared to those in each saline control group, which were significantly increased in IPH and IVH,* p* < 0.05, in IPL and IVL, *p* < 0.01, and in ITL and ITH,* p* < 0.0001. Inflammation was the most serious in intratracheal administration of BLM group. **B** The Neutrophils increased in all groups, compared to those in each saline control group, which were significantly increased in ITH,* p* < 0.001. **C** The Lymphocytes increased in all groups, compared to those in each saline control group, which were significantly increased in ITL,* p* < 0.001. **D** The Monocytes increased in all groups, compared to those in each saline control group, which were significantly increased in ITH, *p* < 0.05, in IPL,* p* < 0.01. (E)The Eosinophils increased in all groups, compared to those in each saline control group, significantly increased in IPL and IVL, *p* < 0.05, in ITL and ITH,* p* < 0.01. **F** The Thrombocytes also increased in all groups, compared to those in each saline control group, which were significantly increased in IPH and IVH, *p* < 0.05, in IPL,* p* < 0.01, and in ITH, *p* < 0.0001

**Fig. 7:**
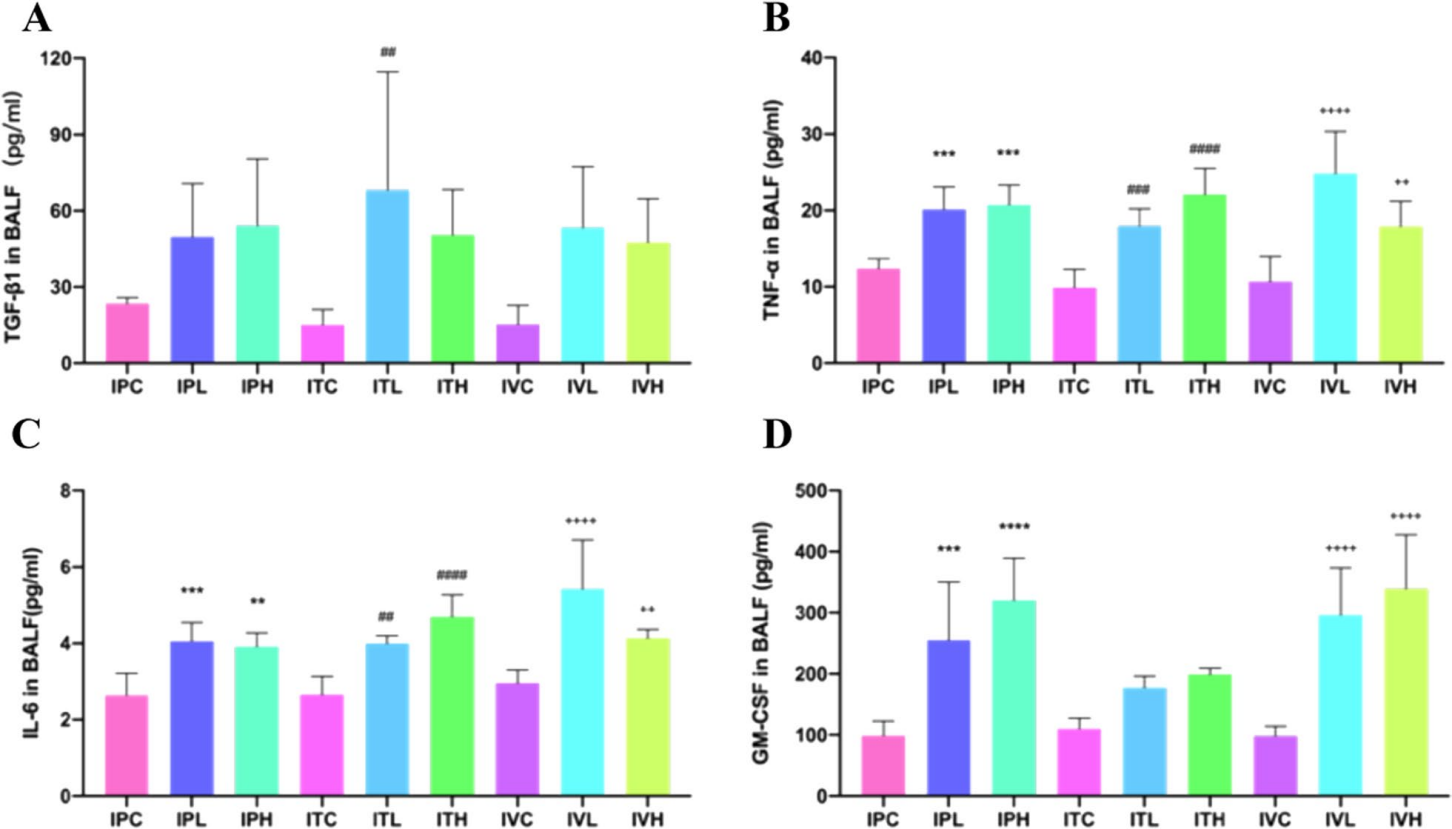
The analysis of BLM-induced PF related proteins in BALF of each group of mice. *p < 0.05, **p < 0.01, ***p < 0.001, ****p < 0.0001, compared to the IPC; ^#^*p* < 0.05, ^##^*p* < 0.01, ^###^*p* < 0.001, ^####^*p* < 0.0001, compared to the ITC; ^+^*p* < 0.05, ^++^*p* < 0.01, ^+++^*p* < 0.001, ^++++^*p* < 0.0001, compared to the IVC. TGF-β1, TNF-α, IL-6 and GM-CSF in the BALF of the mice were increased in all BLM groups than those in each saline control group. **A** TGF-β1 increased in all BLM groups, compared to that in the saline control group, which was significantly increased in ITL, *p* < 0.01. **B** TNF-α increased in all BLM groups, compared to that in each saline control group, which was significantly increased in IVH, *p* < 0.01, in IPL, IPH, and ITL,* p* < 0.001, in ITH and IVL, *p* < 0.0001. **C** The IL-6 showed an up-regulated trend in all BLM groups, compared to that in each saline control, which was significantly increased in IPH, ITL, and IVH, *p* < 0.01, in IPL,* p* < 0.001, and in ITH and IVL, *p* < 0.0001. **D** GM-CSF increased in all BLM groups, compared to that in each saline control, which was significantly increased in IPL,* p* < 0.001, IPH, IVL, and IVH,* p* < 0.0001. TGF-β1, TNF-α, IL-6 and GM-CSF, in the BALF of the mice were significantly different in each BLM administration groups than those in the saline control group

**Fig. 8:**
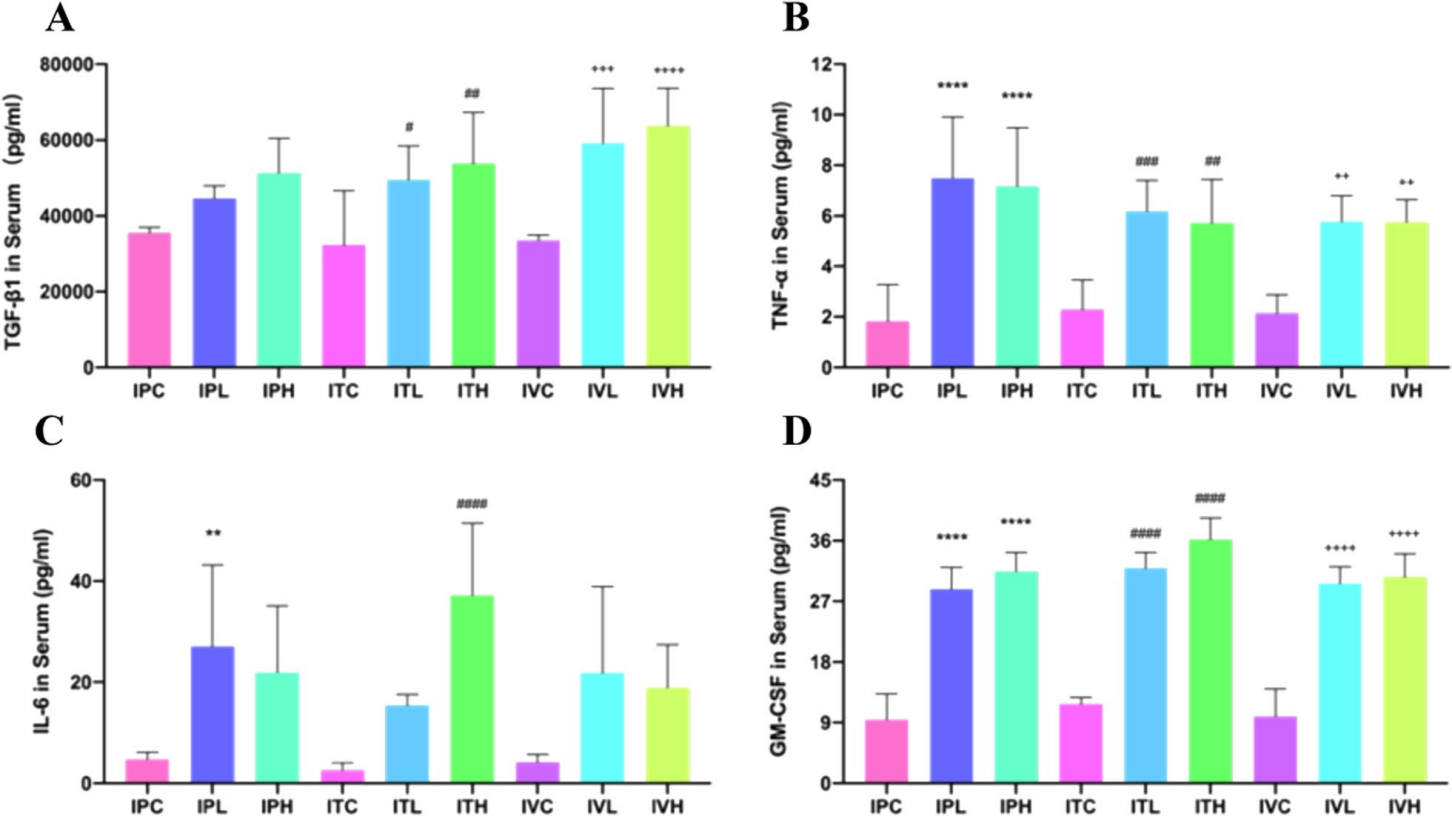
The analysis of BLM-induced PF related proteins in serum of each group of mice. *p < 0.05, **p < 0.01, ***p < 0.001, ****p < 0.0001, compared to the IPC; ^#^*p* < 0.05, ^##^*p* < 0.01, ^###^*p* < 0.001, ^####^*p* < 0.0001, compared to the ITC; ^+^*p* < 0.05, ^++^*p* < 0.01, ^+++^*p* < 0.001, ^++++^*p* < 0.0001, compared to the IVC; TGF-β1, TNF-α, IL-6 and GM-CSF in the serum of the mice were increased in all BLM groups than those in each saline control. **A** TGF-β1 increased in all BLM groups, compared to that in the saline control group, significantly increased in ITL, *p* < 0.05, in ITH,* p* < 0.01, in IVL,* p* < 0.001, and in IVH, *p* < 0.0001. **B** TNF-α increased in all BLM groups, compared to that in the saline control group, which significantly increased in ITH, IVL and IVH, *p* < 0.01, in ITL*, p* < 0.001, and in IPL and IPH, *p* < 0.0001. **C** IL-6 showed an up-regulated trend in all BLM groups, compared to that in the saline control group, which was significantly increased in IPL, *p* < 0.01, in ITH, *p* < 0.0001. Inflammation was the most serious in intratracheal high dose administration of BLM group. **D** GM-CSF also increased in all BLM groups, compared to that in the saline control group, which was significantly increased in all BLM groups, *p* < 0.0001. TGF-β1, TNF-α, IL-6 and GM-CSF in the serum of the mice were significantly different in BLM groups than those in the saline control group

**Fig. 9:**
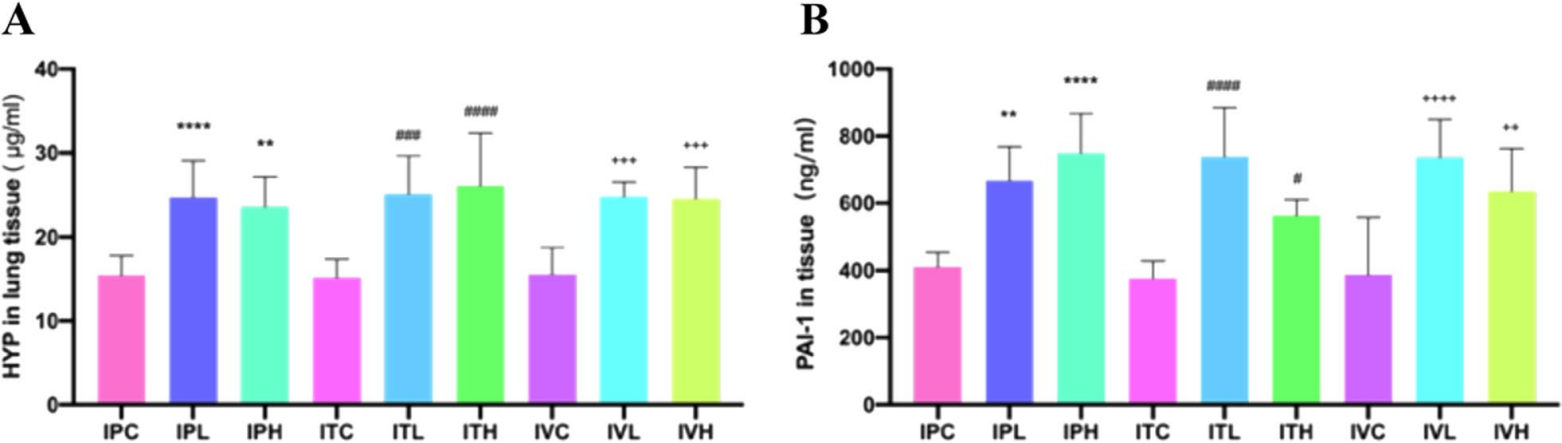
HYP, and PAI-1 changes in Lung tissue. *p < 0.05, **p < 0.01, ***p < 0.001, ****p < 0.0001, compared to the IPC; ^#^*p* < 0.05, ^##^*p* < 0.01, ^###^*p* < 0.001, ^####^*p* < 0.0001, compared to the ITC; ^+^*p* < 0.05, ^++^*p* < 0.01, ^+++^*p* < 0.001, ^++++^*p* < 0.0001, compared to the IVC; HYP and PAI-1 in the lung tissue of the mice were increased in all BLM groups than those in each saline control group. **A** HYP showed an up-regulated trend in all BLM groups, compared to that in the saline control group, which was significantly increased in IPH, *p* < 0.01, in ITL, IVL and IVH, *p* < 0.001, in IPL and ITH, *p* < 0.0001. **B** PAI-1 shows an increased trend in all BLM groups, compared to that in each saline control group, which was significantly increased in ITH, *p* < 0.05, in IPL, and IVH, *p* < 0.01, in IPH, ITL, and IVL, *p* < 0.0001,respectively

The counts of inflammatory cells in BALF of each group were presented in Fig. 6. In this study TGF-β1, TNF-α, IL-6, and GM-CSF antigen in BALF and serum were determined by using theirs ELISA kits; The PAI-1 and HYP antigen in lung tissue were also determined by using ELISA kit. ELISA results showed that compared with that of the saline control group, the total number of BALF cells were significantly different among groups (Fig. 7). Inflammation was most serious in intratracheal administration of the BLM group. TGF-β1, TNF-α, IL-6, and GM-CSF in BALF and serum of the mice were significantly different in BLM groups than those in the saline control groups (Figs. 7 and 8). The contents of HYP and PAI-1 in the lung tissue of the mice in the BLM group were significantly increased compared to those in the saline control group (Fig. 9).

### Masson and PSR staining

**Fig. 10:**
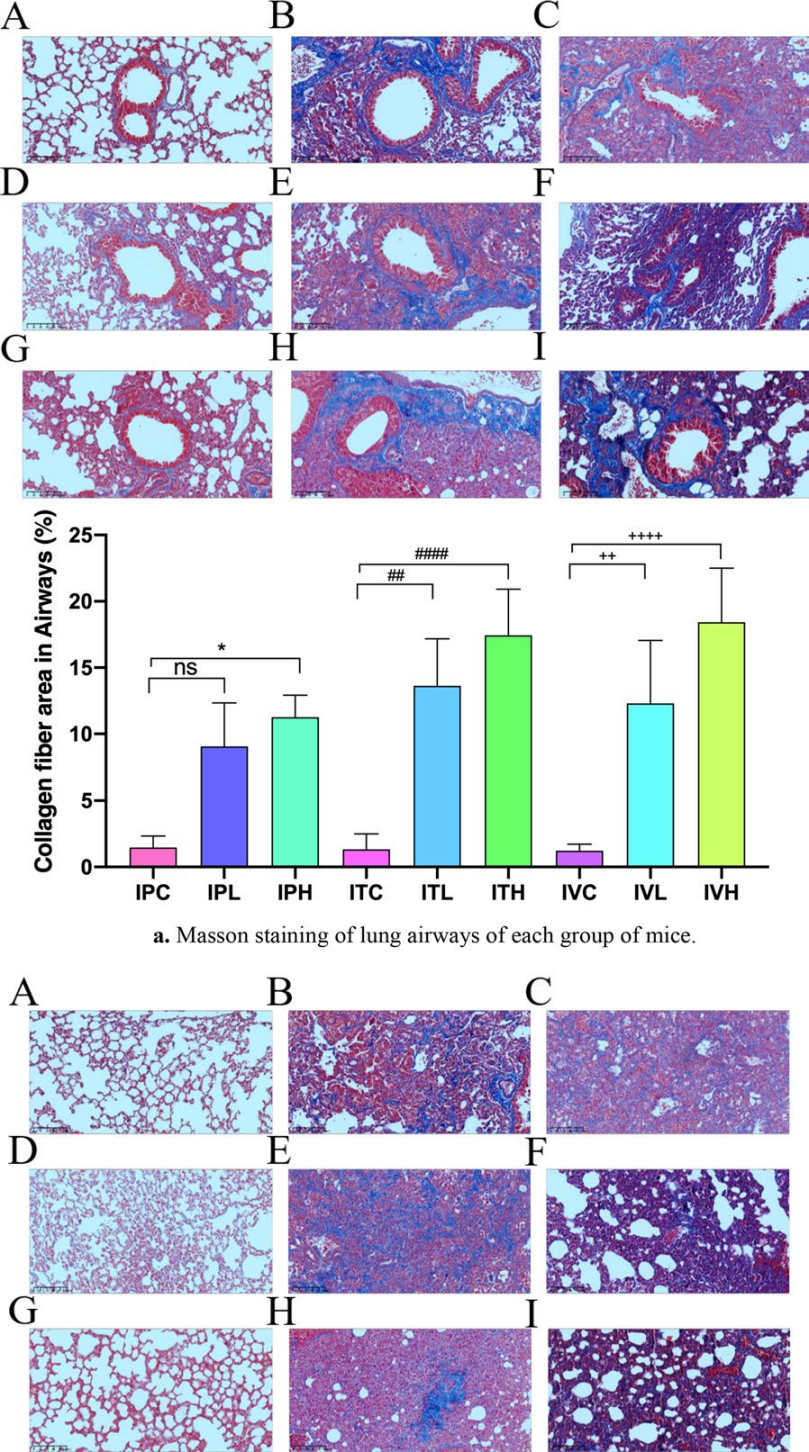

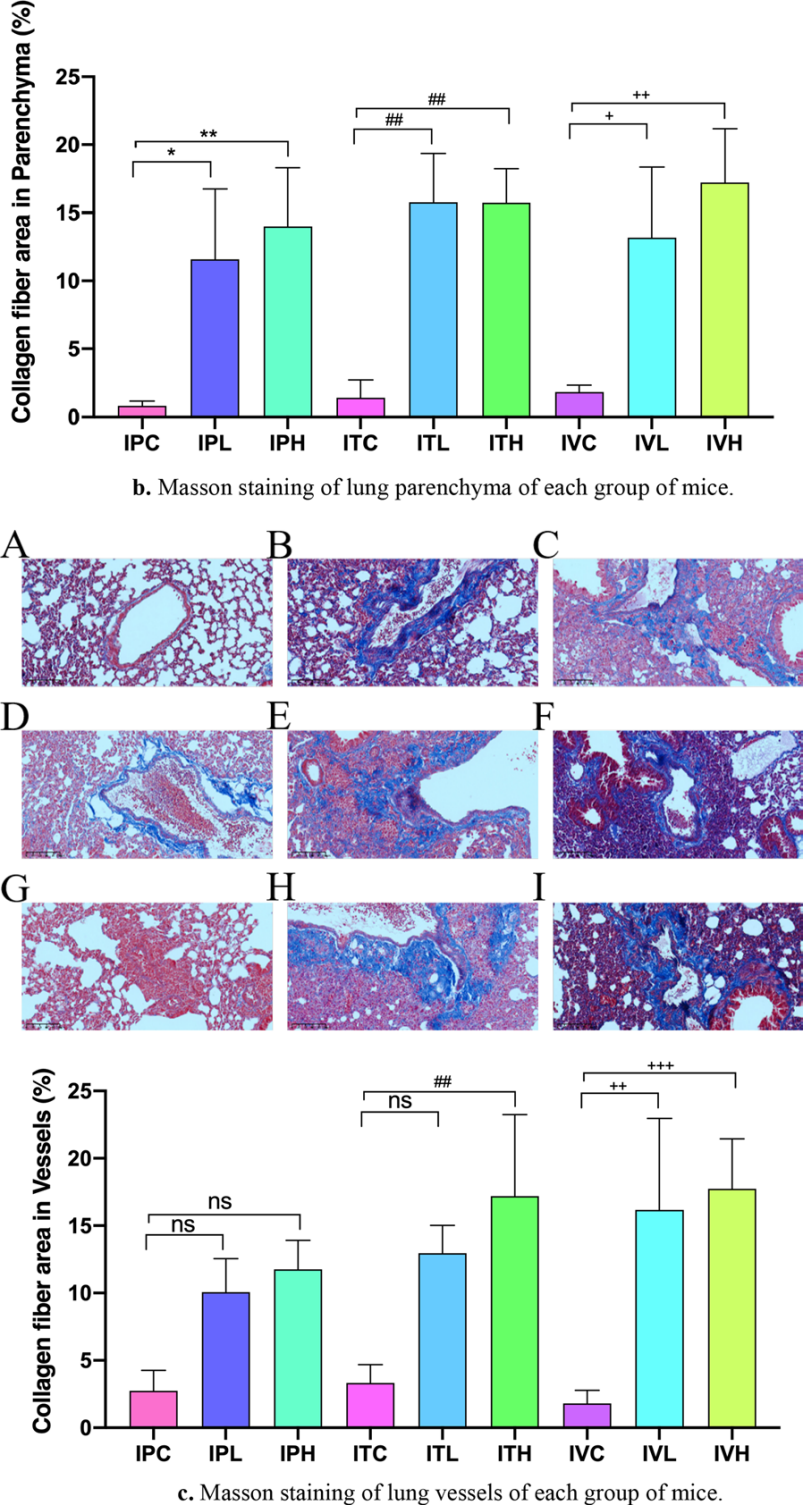
**a** Masson staining of lung airways of each group of mice. Pathological changes of lung Masson's trichrome staining sections, **A**–**I**: × 200. **b** Masson staining of lung parenchyma of each group of mice. Pathological changes of lung Masson's trichrome staining sections, **A**–**I**: × 200. **c** Masson staining of lung vessels of each group of mice. *p < 0.05, **p < 0.01, ***p < 0.001, ****p < 0.0001, compared to the IPC; #*p* < 0.05, ##*p* < 0.01, ###*p* < 0.001, ####*p* < 0.0001, compared to the ITC; +p < 0.05, ++*p* < 0.01, +++*p* < 0.001, ++++ *p* < 0.0001, compared to the IVC; Pathological changes of lung Masson's trichrome staining sections, **A**–**I**: × 200. **A**, **D**, **G** Alveolar structure of the control group normal was shown. **B**, **C** In intraperitoneal injection BLM group, fibrosis was mainly under the pleura, and collagen was evenly distributed in pulmonary interstitial space. **E**, **F** In intratracheal administration BLM group, collagen was located near the bronchia. **H**, **I** In intraperitoneal injection BLM group, fibrosis was mainly under the pleura, and collagen was evenly distributed in pulmonary interstitial space. **J** Masson`s quantification results showed that compare to each saline control, Collagen fiber area significantly increased in all BLM group, *p* < 0.0001. The scale bar is 200 μm

**Fig. 11:**
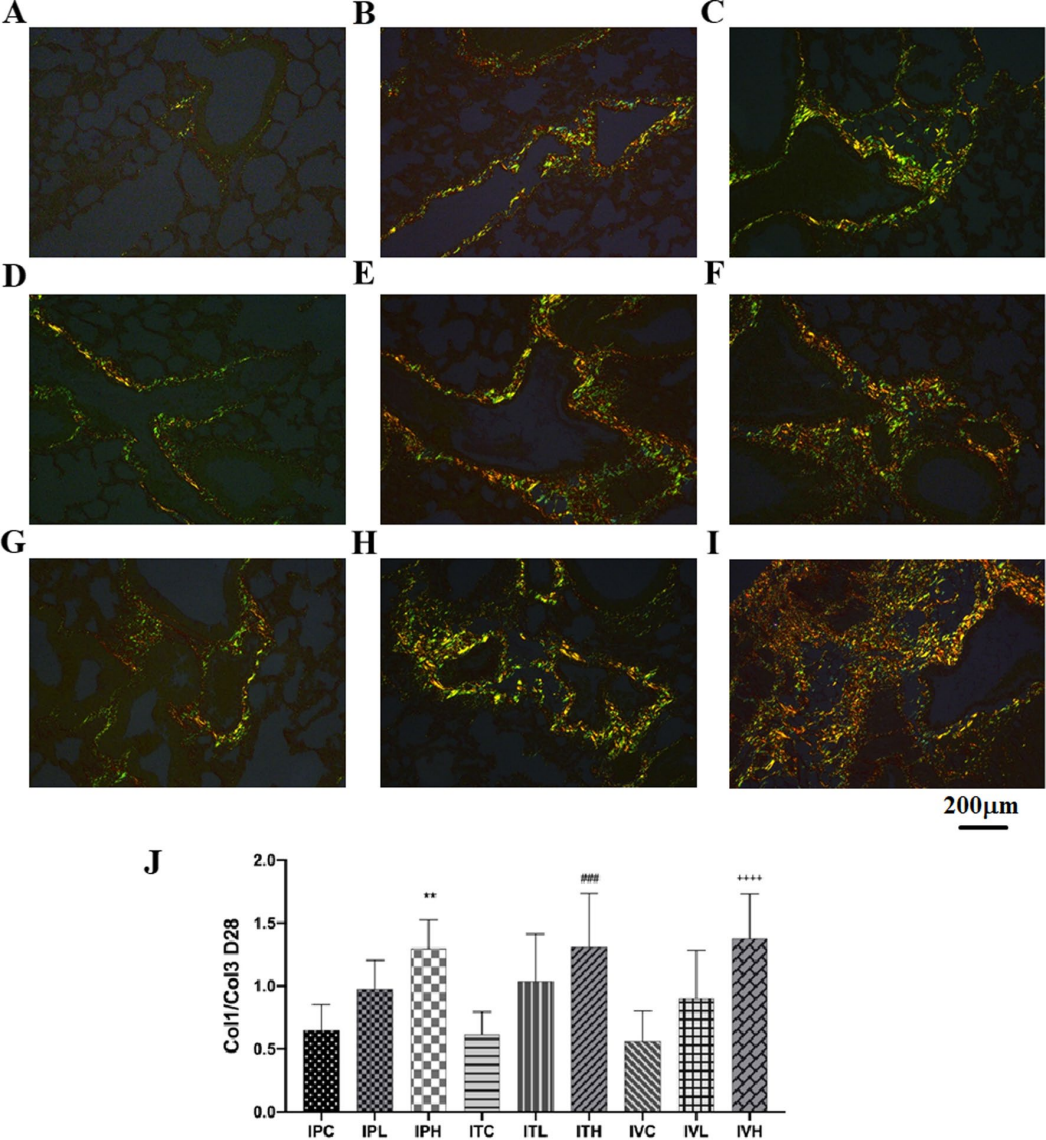
PSR staining of lung tissue of each group of mice. *p < 0.05, **p < 0.01, ***p < 0.001, ****p < 0.0001, compared to the IPC; ^#^*p* < 0.05, ^##^*p* < 0.01, ^###^*p* < 0.001, ^####^*p* < 0.0001, compared to the ITC; ^+^*p* < 0.05, ^++^*p* < 0.01, ^+++^*p* < 0.001, ^++++^*p* < 0.0001, compared to the IVC; Pathological changes of lung PSR staining sections, **A**–**I**: × 200. In PSR staining Col 1 showed orange/bright red, while Col 3 showed green. **A**, **D**, **G** Control group normal alveolar structure was shown; **B**, **C** In intraperitoneal injection group, fibrosis was mainly under the pleura, and collagen was evenly distributed in pulmonary interstitial space. **E**, **F** In intratracheal administration group, collagen was located near the bronchia. **H**, **I** In intraperitoneal injection group, fibrosis was mainly under the pleura, and collagen was evenly distributed in pulmonary interstitial space. PSR staining results were also consistent with the results of HE and Masson staining. Compared with that of the saline treatment group, the ratio of Col 1/Col 3 was significantly increased. IPH, ITH, and IVH were significantly increased compared to those in the saline treatment group. IPH (*p* < 0.01), ITH (*p*  < 0.001), and IVH were significantly increased (*p*  < 0.0001). The scale bar is 200 μm

Masson staining results showed that a small amount of light blue matrix was deposited around blood vessels and bronchus in saline control group, but no obvious collagen components were seen in the alveoli and pulmonary septum (Fig. 10a–c). 2–3 mice in each group showed mild alveolitis, with a slight thickening of the alveolar septum and slight congestion of blood vessels. 10A, 10D, 10G: Alveolar structure of the control group normal was shown. 10B, 10C: In intraperitoneal injection BLM group, fibrosis was mainly under the pleura, and collagen was evenly distributed in pulmonary interstitial space. 10E, 10F: In intratracheal administration BLM group, collagen was located near the bronchia. 10H, 10I: In intraperitoneal injection BLM group, fibrosis was mainly under the pleura, and collagen was evenly distributed in pulmonary interstitial space. 10 J: Masson`s quantification results showed that compared to each saline control, Collagen fiber area significantly increased in all BLM group (Fig. 10a–c), *p* < 0.05. PSR staining was consistent with the results of Masson staining. Compared with the saline treatment group, the ratio of Col 1/Col 3 was significantly increased. IPH, ITH, and IVH were significantly increased compared to those of the saline treatment group (Fig. 11A–J).

### Ultrastructure image of TEM

**Fig. 12:**
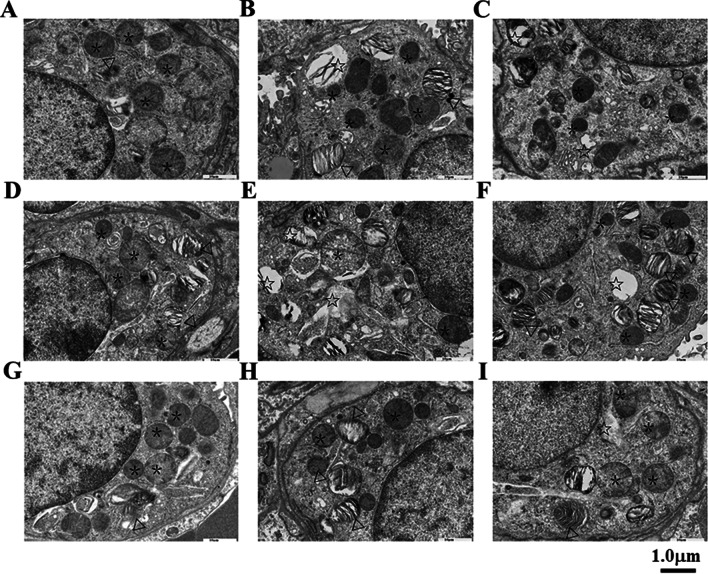
TEM Ultrastructure images of lung tissue in each group of mice. Pathological changes of lung TEM images. **A**–**I** × 8000. **A**, **D**, **G** The saline control group had no pathological changes, normal organelle structure, lamellar bodies (white triangle▽), and phagosome (*) were shown in alveolar type II cells. In the intraperitoneal injection group, **B**, **C** Type II alveolar epithelial cell proliferation, and the vacuole-like transformation of lamellar corpuscles (white star☆) were shown in the cell. **E**, **F** in Intratracheal administration group, type II alveolar epithelial cell proliferation, dysmorphic lamellar bodies, and the vacuole-like transformation of lamellar corpuscles (white star☆) were shown in the cell. **H**, **I** In tail vein injection group, morphological variation, type II alveolar epithelial cell proliferation, dysmorphic lamellar bodies, and the vacuole-like transformation of lamellar corpuscles (white star☆) were shown in the cell. Scale bar is 1.0 μm

TEM images found type I alveolar epithelial cell degeneration, disintegration and shedding, endothelial cell swelling, type II alveolar epithelial cell proliferation, abundant microvilli on the free surface of the cell, vacuole-like transformation of lamellar corpuscles in the cell, irregular nucleus morphology, partly visible Jagged bumps. The base membrane was thickened, partly thinner, uneven, and integrity was destroyed. The interstitial fibroblasts increased, the intracellular procollagen increased, the rough endoplasmic reticulum expanded, and the collagen fibers were stacked in a large amount in the interstitial, and which were arranged in a crisscross pattern. Alveolar septum macrophages increased. See Fig. 12.

## Discussion

BLM is clinically used to treat a variety of neoplastic diseases, and its main adverse reaction is to induce severe PF [27]. In 1970, some scholars tried BLM in the treatment of 237 cancer patients and found that BLM would damage the skin, lungs, and mucous membranes [28]. In 1972, Halnan et al. [29] used 103 cases of cancer patients treated by BLM to undergo clinical observation, with one patient died of pneumonia. In 1973, Bedrossian et al. [30] performed an electron microscope observation on ultra-thin sections of lung tissue of patients who died of BLM. The results showed that type I alveolar epithelial cells decreased, and type II alveolar epithelial cells proliferated, and vascular endothelial cells and immune cells were damaged. Infiltration of alveoli, large amounts of collagen deposition, interstitial edema, diffuse alveolar, and pleural fibrosis, etc., were similar to clinical IPF lesions found in the past. In 1974, Adamson et al. [31] first adopted repeated intraperitoneal injections of BLM. An animal model method of rat lung interstitial fibrosis was established. Because BLM-induced pulmonary fibrosis (bleomycin-induced pulmonary fibrosis, BPF) has the most similar histopathological changes to human PF. The animal model of BLM replication is currently the most popular method for studying PF.

To examine specific mechanisms and processes involved in the development of pulmonary fibrosis, the mode of the BLM administration is very important. The drug can be administrated intraperitoneally, intravenously, subcutaneously, or intratracheally but intravenous (iv) and intratracheal (it) which are the more commonly used routes [27]. The iv administration (20 mg/Kg, twice a week for 4–8 weeks) closely mimics the way that humans are exposed to a drug regimen during chemotherapeutic treatment. Initially, the damage is limited to the cells of the lung interstitial and it can include signs of acute lung injury (damage to the alveolar epithelium, leakage of fluid and plasma proteins into the alveolar space, alveolar consolidation, and the formation of hyaline membranes). Focal necrosis of type I epithelial cells and the induction of metaplasia in type II epithelial cells are also present, along with inflammatory infiltrates and fibrosis in sub-pleural regions. Unfortunately, this method of administration is not able to guarantee the full development of fibrosis in all animals; the time-lapse for disease development is relatively long, being the initial lesions at the epithelial level.

At present, the more mature method recognized is transtracheal administration [32, 33]. Most of the animals used are rats and mice. The common supplies are C57BL/6 mice, SD rats, and Wistar rats. The pathological changes caused by the model are similar to those in the clinic. The drug enters the lungs through tracheal perfusion or atomization inhalation. The former has higher technical requirements, while the latter makes the drug evenly distributed in the body, but the model requires special equipment and is less used. Tracheal perfusion administration requires shorter anesthesia time than rapid nasal administration, which is simple and fast, causing less animal damage, but requiring high of technical proficiency for the operator. Intraperitoneal injection administration allows BLM to absorb blood from the abdominal cavity to the lungs, playing a role in producing IPF. The use of this method of the model can reduce the degree of fibrosis caused by differences in surgical operations, with simple operation, low animal mortality, the more uniform distribution of lesions, but the number of administrations and the doses are larger. The modeling time is longer, with high cost, and it has certain limitations [34]; tail vein injection is mainly used for the preparation of mouse IPF models.

In BPF animal models, its typical feature is alveolar epithelial cell damage. The early-stage manifests as acute inflammation, including alveolar epithelial cell injury, inflammatory cell infiltration, and the release of inflammatory mediators. In the subacute stage, the proliferation and differentiation of fibroblasts and the expression of pro-fibrotic cytokines will appear in the lesion area; there will be a large amount of extracellular matrix deposition and fibrotic lesions [35]. The BPF mouse model and found that the lungs of the model group showed varying degrees of alveolar structural damage, inflammatory cell infiltration, and a large amount of collagen-based extracellular matrix deposition [36]. The gene expression test results showed that mouse lung tissue interleukin, collagen gene connective tissue growth factor, and transforming growth factor were all increased to varying degrees compared with those in the normal control group, which was consistent with the histopathological changes of IPF.

The lesion site of the BPF animal model will be different due to different administration methods. The PF lesions formed by the tracheal administration model are mainly distributed around the bronchi and bronchioles, especially the hilar, which is different from the clinical lesions [37]. Tail vein injection simulates the administration of BLM in humans. The pathology and related cytokine changes are similar to clinical IPF [38]. Intraperitoneal injection of BLM mice model, leads to inflammation around the bronchus in the early stage, with fibrotic lesions, mid-stage inflammation, and inflammation. The fibrosis area is mainly concentrated under the pleura, while the subpleural fibrosis area is enlarged in the later period. Compared with tracheal administration, it is closer to the imaging manifestations of human IPF and is similar to the pathological distribution of clinical IPF [39].

Bleomycin can induce inflammation and fibrosis in the lungs of experimental animals in a short period. In this experiment, both intratracheal administration and tail vein injection observed a strong inflammatory response, manifesting as many inflammatory cells as possible. With lung infiltration, the cell count and protein content in BALF increased significantly. This study also found that the inflammatory response in the lungs of ITL, ITH model group was stronger than that in IVL and IVH model group, and their BALF protein contents were significantly higher than those of ITL and ITH model groups.

The measurement of lung tissue HYP, lung interstitial injury index, and other indicators that reflected the degree of pulmonary fibrosis showed that both animal models could better present the occurrence of pulmonary fibrosis. Indicators were compared between two BLM groups with IVL and IVH groups slightly higher than ITL and ITH groups, but there was no significant difference between these two. Facial pathological observation revealed that the occurrence of pulmonary fibrosis after tail vein injection was concentrated under the pleura and evenly distributed in the interstitial of the lungs, while intratracheal drugs were concentrated around the trachea and distributed unevenly in different lung lobes. Therefore, the pathology and characteristics of IVL and IVH model groups were more in line with IPF [40, 41].

In this study, the none mortality rate of mice in intraperitoneal injection, and intravenous administration of BLM group, indicates that the mice have a better tolerance to small doses of multiple administration BLM. In IVL and IVH model groups the main adverse reactions were the injection site, namely the tail swelling of necrosis and gastrointestinal reactions. The main complication of tracheal injection of BLM was severe inflammation in the early lungs. Therefore, the low-dose multiple tail vein injection of BLM pulmonary fibrosis model had simple methods, easy control of experimental conditions, good reproducibility. Many disease models could be induced in a short time, low mortality, and fibrotic lesions were evenly distributed under the pleura, etc. Characteristics, worthy of promotion, could be used for research on the pathogenesis of pulmonary fibrosis and observation of the efficacy of anti-fibrotic drugs. In addition, C57/BL6 mice were selected for this experiment not only because of their high sensitivity to BLM drugs but also because C57/BL6 mice were slightly larger than white mice, which were suitable for multiple tail vein injections.

## Conclusions

Based on the comparison of three different methods of animal model construction, high doses of them overall showed more compliable. BLM could successfully induce animal models of pulmonary fibrosis, but there were certain differences in the fibrosis formation sites of them three, and tail vein injection of BLM induced pulmonary fibrosis model was closer to idiopathic pulmonary interstitial fibrosis.

## Supplementary Information

**Additional file 1.** Animal procedure ethical approvement by the Committee on the Ethics of Animal Experiments of Fudan University.

## Data Availability

The data analyzed during the current study are available from the corresponding author on reasonable request.

## Abbreviations

3DROI: 3D regions of interest
18F-FDG PET/CT: Positron emission tomography with 2-deoxy-2-[fluorine-18] fluoro-d-glucose integrated with computed tomography
BALF: Bronchoalveolar lavage fluid
BLM: Bleomycin
BPF: Bleomycin-induced pulmonary fibrosis
Cchord: Chord compliance, between 0 and 10 cm H_2_O
ELISA: Enzyme linked immunosorbent assay
FVC: Forced vital capacity
FRC: Functional residual capacity
GM-CSF: Granulocyte–macrophage colony-stimulating factor
HYP: Hydroxyproline
IL-6: Interleukin-6
IPF: Idiopathic pulmonary fibrosis
ITL: Intratracheal administration of BLM 3 mg/kg group
ITH: Intratracheal administration of BLM 5 mg/kg group
ITC: Saline intratracheal administration control group
IPL: Intraperitoneal injection of BLM 20 mg/kg group
IPH: Intraperitoneal injection of BLM 50 mg/kg group
IPC: Saline intraperitoneal control group
IVL: Ttail vein injection of BLM 10 mg/kg group
IVH: Tail vein injection of BLM 20 mg/kg group
IVC: Saline tail vein injection control group
MLD: Mean lung density
PAI-1: Plasminogen activator inhibitor-1
PET/CT: Positron emission tomography/computed tomography
PEF: Peak expiratory flow
PFT: Pulmonary function testing
PSR: Picro-Sirius red staining
TEM: Transmission electron microscopy
TGF-α: Transforming growth factor alpha
TGF-β1: Transforming growth factor beta 1
