# Supplementary material for: Pulmonary fibrosis model of mice induced by different administration methods of bleomycin
Source: BMC Pulm Med. 2023 Mar 21;23:91. doi: 10.1186/s12890-023-02349-z (PMC10029181; doi:10.1186/s12890-023-02349-z)
Supplement: Supplementary file 1 — Additional file 1. Animal procedure ethical approvement by the Committee on the Ethics of Animal Experiments of Fudan University. [file 12890_2023_2349_MOESM1_ESM.pdf]

复旦大学药学院实验动物伦理委员会

研究项目伦理审批件

伦药批第（2021-01-HSYY-DJC-002）号

<table>
<tr><td>项目名称</td><td colspan="5">博莱霉素不同给药方法致小鼠肺纤维化模型的建立</td></tr>
<tr><td>项目类别</td><td colspan="5">基础＿√＿　临床＿＿＿　药物＿＿＿</td></tr>
<tr><td>项目来源</td><td colspan="5">自筹</td></tr>
<tr><td>申办单位</td><td colspan="3">复旦大学附属华山医院</td><td>单位负责人</td><td>毛颖</td></tr>
<tr><td>研究部门</td><td>中西医结合研究院</td><td>项目负责人</td><td>董竞成</td><td>职　称</td><td>教授</td></tr>
<tr><td colspan="6">伦理审查意见</td></tr>
<tr><td colspan="4">Δ 同意</td><td colspan="2">√</td></tr>
<tr><td colspan="4">Δ 修改后同意</td><td colspan="2"></td></tr>
<tr><td colspan="4">Δ 不同意（项目终止或暂停）</td><td colspan="2"></td></tr>
<tr><td colspan="6">审批意见
该项目方案已通过实验动物伦理审查，同意实施。

主任委员（签名）＿＿＿＿＿　　复旦大学药学院实验动物伦理委员会
（盖章）

2021年01月18日</td></tr>
</table>
